# Research in progress—LungSEARCH: a randomised controlled trial of surveillance for the early detection of lung cancer in a high-risk group

**DOI:** 10.1136/thoraxjnl-2015-207433

**Published:** 2015-07-02

**Authors:** Stephen G Spiro, Allan Hackshaw

**Affiliations:** 1 Department of Thoracic Medicine, University College London Hospitals, and The Royal Brompton Hospital, London, UK; 2 Deputy Director Clinical Trials Unit, University College London, London, UK

**Keywords:** Lung Cancer

## Abstract

Low-dose CT screening for lung cancer is effective but expensive. Therefore, cheaper or more focused screening strategies may be required. LungSEARCH is a randomised prospective trial of 1568 high-risk individuals (ie, current or former moderate to heavy smokers with mild/moderate COPD) who undergo either annual sputum cytology/cytometry testing or no screening. Those with abnormal sputum then receive annual CT and fluorescent bronchoscopy for the remainder of 5 years, to identify early stage lung cancer. It is hoped that these simple initial tests could identify those requiring expensive CT scans, and the aim is to demonstrate a stage shift towards early stage cancers.

Trial registration numbers ISRCTN: ISRCTN80745975, clinicaltrials.gov: NCT00512746.

## Introduction

The majority of patients with lung cancer (80%) have advanced disease at the time of diagnosis, making them ineligible for curative treatment, which contributes to the low overall 5-year survival (around 7% in the UK). By contrast, the prospects for patients with early stage disease are much more favourable, with a 5-year survival of 60%–70% among those with stage Ia non-small cell lung cancer.

In the 1970s screening for lung cancer with chest radiography identified more cancers than in the control groups, but there was no improvement in mortality from the randomised trials. In 2006, several studies of CT scanning were used to generate hypotheses after identifying peripheral adenocarcinomas in prevalence screens, with the numbers discovered varying by population and whether the study group were Asian, where peripheral adenocarcinomas are more common.

## Recent or ongoing lung cancer screening trials

The National Lung Cancer Screening Trial (NLST), in the USA, was the first large randomised study to show that low-dose CT can identify lung cancers at an earlier stage in an unselected smoking population and, importantly, reduce mortality.^1^ The trial assigned 53 452 heavy smokers aged between 55 and 74 years to either three annual CT screens or chest radiographs. It showed a statistically significant 20% reduction in mortality from lung cancer with CT. However, CT screening is expensive and a cost-effectiveness analysis based on the NLST results suggested that the cost per quality adjusted life year could be as high as US$81 000.^2^

The results on mortality from the NELSON trial (conducted in Belgium and The Netherlands) are due in 2015–2016, and are expected to confirm the findings of the NLST. Eligible participants were aged 50–75 years, and moderate to heavy current or former smokers (who had quit less than 10 years before). 15 822 participants were enrolled of whom 7915 were assigned to low-dose CT screening at baseline, then at years 2 and 4; and 7907 individuals had no screening (only smoking cessation). Interim results indicate a high cancer detection rate (85%) and low false-positive rate (1.4%) after three screening rounds combined and 2-year follow-up; with a positive predictive value of 40%.^3^

There are ongoing attempts to use CT more efficiently (including the LungSEARCH trial outlined below), or biomarkers. The UK Lung Screening randomised trial uses a validated Liverpool Lung Project risk model to identify individuals who have a risk of at least 5% of developing lung cancer in the next 5 years, and only these are invited to participate. They are then randomised to receive either a single (prevalence) CT scan, or no screening.^4^ Another ongoing trial (in Scotland) is using a set of immune antibody markers to identify those at high risk of lung cancer and then follow these subjects closely with CT.

## Lung cancer risk in smokers with COPD

The first National Health and Nutrition Examination Survey showed that individuals with COPD were significantly more likely to develop lung cancer than those with normal lung function.^5^ The cumulative risk of developing incident lung cancers in patients with moderate COPD was 4% after 5 years, and several other studies have shown a significant association between impaired lung function and lung cancer risk. It is also known that patients with COPD (of all grades of severity) are at increased risk of developing lung cancer if they have abnormal sputum cytology. These findings were used to help justify the LungSEARCH trial.

## Rationale for LungSEARCH

Targeting high-risk subjects should increase the yield of cancers, making it more cost-effective. COPD represents an additional risk for developing lung cancer, as are previous head and neck cancers, asbestos exposure and more advanced age.

When launched, LungSEARCH was the first trial to focus on high-risk individuals and to use an initial cheaper screen to identify those who may have predisposition to lung cancer or have severe dysplasia/carcinoma in situ and allow detailed investigation.

In the UK, squamous cell tumours were prevalent, and since most of these arise from central airways, we wished to re-explore the value of sputum cytology plus cytometry as a tool to identify malignant cells and also dysplastic cells, which could be precursors of malignancy. There was evidence to suggest that the sensitivity of sputum screening may be significantly enhanced by the application of computer-assisted image analysis (automated image cytometry) to quantitatively analyse the nuclear structure and DNA content of individual cells.

## LungSEARCH study design

Eligible participants were predominantly well but had mild to moderate COPD (using the Global Initiative for Chronic Obstructive Lung Disease criteria), and were also either current smokers with ≥20 pack years smoking history and/or 20-year duration of smoking, or former smokers who had quit within 8 years with ≥20 pack years smoking history and/or 20-year duration of smoking.

**Figure 1:**
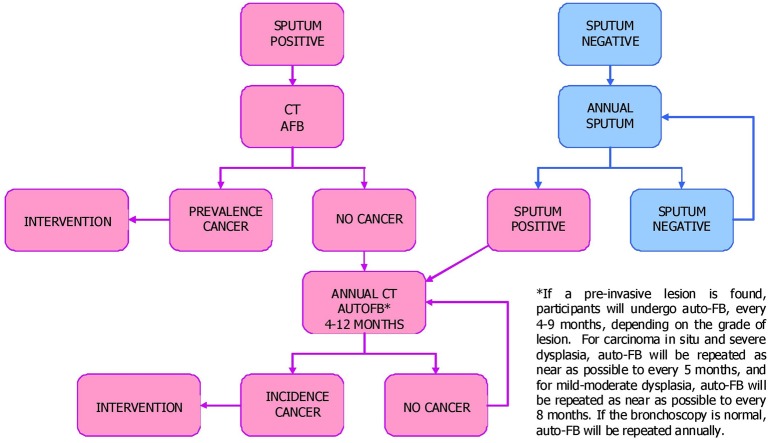
Flow diagram showing what happens to individuals in the screened (surveillance) arm of the LungSEARCH trial. AFB, autofluorescence bronchoscopy.

Participants were randomised to the control group (no intervention) or annual screening for 5 years. The screened group were asked to provide early morning sputum as the initial screen, which is a cheap and accessible test. All those with abnormal sputum results (positive cytology or cytometry) would then be offered surveillance for up to 5 years using an annual CT scan and annual autofluorescence bronchoscopy (AFB); the pathway is summarised in figure 1. Those with normal sputum results would provide another sample yearly, unless they have an abnormal result. The majority of published studies on AFB have shown a significant increase in diagnostic sensitivity for dysplasia and carcinoma in situ. There is also evidence to suggest that the detection of invasive carcinoma is enhanced by fluorescence bronchoscopy and this technique has been used to assess patients with abnormal sputum cytology. The clinicians in each participating centre would decide from any abnormal bronchoscopic and/or CT appearances whether follow-up should be more than annual.

The control subjects were not contacted after randomisation and are only expected to have an exit chest X-ray at the end of 5 years. Any cancers or deaths would be identified through patient records or the national cancer registry.

The trial is powered to detect a stage shift between the screened and control groups; specifically to show that the proportion of cancers that are early stage (I and II) is 50% in the surveillance group and only 10% in the control group; and for this we require at least 74 lung cancers.

The screened group were also asked to participate in Lung reSEARCH, where annual blood and sputum would be collected and stored in a central tissue bank at Leeds University. All those undergoing AFB would also be asked to consent to provide control samples of bronchial mucosa as well as two samples from any suspicious area, in addition to those samples taken for pathology. We therefore have the opportunity to follow the natural history of dysplastic lesions for up to 5 years, providing an important contribution on the natural history of dysplastic lesions, whose management remains controversial.

## Progress

The study recruited 1568 individuals between August 2007 and March 2011 (785 in the screened group and 783 controls). Follow-up finishes in March 2016. Ten centres across the UK were involved, with COPD patients approached from either the hospital clinics or local general practitioners (GP) practices. The centres were in London (Royal Brompton, Chelsea and Westminster, and University College London Hospital), Leeds, Cambridge, Leicester, Manchester, Sunderland, Coventry and Belfast. Recruitment from general practices proved much more difficult than expected, due mainly to reluctance of the majority of practices to participate in research. Many demanded payment for access and a separate grant was obtained to provide funds for this. The level of GP co-operation was lowest in London.

As of April 2015, 65 lung cancers have been found through screening, patient medical records or the national cancer registry. Initial results showed an incidence of abnormal sputum (using either cytology or cytometry) of 23% of those in the screened arm in the first year, and 15% in the second year (excluding those who had a previous abnormal sputum).

The study will inform on the value of a simple initial screen in a high-risk population, the value of annual CT surveillance in those with sputum dysplasia, the role of annual AFB, the effect of smoking cessation in this high-risk group and cost-effectiveness of this screening policy.

If sputum testing in high-risk individuals identifies a group especially at risk of lung cancer, whose tumour is discovered during surveillance, and the study demonstrates a stage shift between the control and the active group, then a larger study looking at mortality could be performed to confirm the hypothesis generated in this study.
